# Dietary Bean Consumption and Human Health

**DOI:** 10.3390/nu11123074

**Published:** 2019-12-17

**Authors:** Henry J. Thompson

**Affiliations:** Cancer Prevention Laboratory, Colorado State University, Fort Collins, CO 80523, USA; henry.thompson@colostate.edu; Tel.: +1-970-491-7748; Fax: +1-970-491-3542

The focus of this Special Issue is on grain legumes, which are commonly referred to as pulses [1]. Pulses have been a key staple food crop, selected by the inhabitants of most agricultural centers of domestication between 8000 to 10,000 years ago as a primary source of protein and calories [2]. However, in the last 100 years, pulses have been abandoned as a staple food and relegated to occasional consumption as vegetables [3]. When a decline in pulse consumption occurs in a population, there is a temporal proximity to a rise in chronic disease rates [4,5]. While it is commonly asserted that a return to pulse consumption would be good for the health of the general population and for planetary health [6,7], there are many skeptics that insist that the evidence supporting this claim is limited.

Since the World Health Organization (WHO)/Food and Agriculture Organization (FAO) International Year of the Pulse in 2016 [8], there has been an uptick in the number of pulse-related publications across many disciplines. In the journal *Nutrients*, 20 papers have been published in the last two years (2018 and 2019). Eight of these papers are collected in this Special Issue. In order to guide the reader interested in the topic of pulses and human health, the references to all 20 papers are provided [9,10,11,12,13,14,15,16,17,18,19,20,21,22,23,24,25,26,27,28]. These publications can be grouped into three primary domains that are judged to be critical to understanding the multi-dimensional framework underlying pulse consumption and human health. Each domain is briefly discussed, with a concise notation on each contribution in this Special Issue according to the domain to which it was assigned.

## 1. Domain: Germplasm Resources

Created equal? Common characteristics and distinct traits. The FAO of the WHO currently lists 16 edible pulses [29]. Of these, the production and consumption of four pulses are globally predominant, common bean (*Phaseolus vulgaris*, L.), chickpea (*Cicer arietinum* L.), dry pea (*Pisum sativum* L.), and lentil (*Lens culinaris* L.) [30]. From a human health perspective, commonalities among these pulses include high protein and dietary fiber contents with very low levels of dietary lipid. However, there is considerable variability among these pulse crops in qualitative aspects of protein and fiber as well as significant variability in their small-molecule profiles [29]. Even within a specific pulse crops, e.g., common bean, there is significant variation among market classes (genetically distinct groupings of cultivars that have trait-specific economic value), and measurable variation is also noted among cultivars within a market class [31,32,33]. While it can be argued that complex situations need to be simplified for consumer adoption, it is essential that differences among and within pulse crops be recognized and understood relative to human health potential. The Special Issue features three papers in this domain. Two papers describe the potential health-related traits of two common bean market classes, i.e., mung bean [17] and lupin bean [23]. The genetic basis for variation among snap beans, the edible immature stage of common bean, that regulates the content of phenolic compounds is also reported [21]. Of the other 12 pulse-related papers published by *Nutrients* but not included in this Special Issue, none were placed in this domain.

## 2. Domain: Health Benefits

What are the benefits of pulses for human health? There are two broad areas into which the effects of pulses on human health can be divided, that related to the provision of phytochemicals classified as nutrients and that related to other chemical constituents that may have effects either on physiological function, e.g., gut health, or on various diseases, e.g., chronic disease risk. Two papers in the Special Issue considered the nutritional aspects of bean (iron [24] and protein [18]), and others focused on gut health [16] and chronic disease (obesity) [22]. Of the other 12 papers not in this Special Issue, 9 were placed in this domain, and the topics covered included iron nutrition [12,15,25], effects on muscle strength [9], gut health [10,11,19], glycemic index [28], and metabolic syndrome [20].

## 3. Domain: Consumption

Pulse consumption can be viewed as a lifestyle behavior [34,35]. Given the conclusions from work reported in relation to the International Year of the Pulse that there is significant merit to increasing pulse consumption, an understanding of consumer knowledge, attitudes, and habits is essential to developing a lifestyle change strategy. One paper on this topic is presented in this Special Issue, considering an Australian cohort [14]. Of the other papers not in this Special Issue, 3 were placed in this domain. They examined knowledge, attitudes, and/or habits in adults [13], registered dietitians [26], and Hispanic and non-Hispanic populations living in the mid-west region of the United States [27].

## 4. Concluding Comment

While pulse crops are ancient foods, there is a great deal that can be learned about the potential for these grains to promote human health and wellbeing. It is critical to make distinctions between pulses and other edible legumes such as oil seed legumes, e.g., peanuts and soy bean, in order to maximize consumer awareness and the understanding of pulses as an important but neglected staple food crop for 21st century challenges related to human and planetary health.
